# Resolving cellular systems by ultra-sensitive and economical single-cell transcriptome filtering

**DOI:** 10.1016/j.isci.2021.102147

**Published:** 2021-02-05

**Authors:** Andres F. Vallejo, James Davies, Amit Grover, Ching-Hsuan Tsai, Robert Jepras, Marta E. Polak, Jonathan West

**Affiliations:** 1 Clinical and Experimental Sciences, Sir Henry Wellcome Laboratories, Faculty of Medicine, University of Southampton, SO16 6YD Southampton, UK; 2 GlaxoSmithKline, Gunnels Wood Road, Stevenage SG1 2NY, UK; 3 GlaxoSmithKline, 200 Cambridge Park Drive, Cambridge, MA 02140, USA; 4 Institute for Life Sciences, University of Southampton, Southampton SO17 1BJ, UK; 5 Cancer Sciences, Faculty of Medicine, University of Southampton, Southampton, UK

**Keywords:** Biological Sciences, Omics, Transcriptomics

## Abstract

Single-cell transcriptomics suffer from sensitivity limits that restrict low abundance transcript identification, affects clustering and can hamper downstream analyses. Here, we describe Constellation sequencing (Constellation-Seq), a molecular transcriptome filter that delivers two orders of magnitude sensitivity gains by maximizing read utility while reducing the data sparsity and sequencing costs. The technique reliably measures changes in gene expression and was demonstrated by resolving rare dendritic cell populations from a peripheral blood mononuclear cell sample sample and exploring their biology with extreme resolution. The simple and powerful method is fully compatible with standard scRNA-Seq library preparation protocols and can be used for hypothesis testing, marker validation or investigating pathways.

## Highlights

• Constellation-Seq addresses the issues of sensitivity and sparsity in scRNA-sequencing
• Constellation-Seq can be used with DropSeq and chromium 3′ chemistry
• Constellation-Seq resolves rare dendritic cell populations from PBMC

## Graphical abstract

**Figure:**
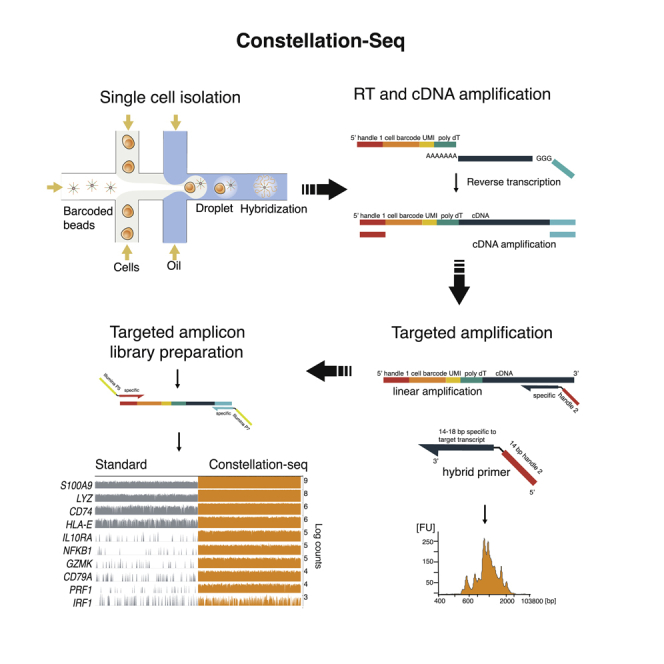

## Introduction

The dramatic uptake and expansion of single-cell transcriptome analysis tools has transformed biological research, enabling reconstruction of population architectures and underlying processes to be revealed. The tools rely on compartmentalization of single cells with the introduction of unique genetic barcodes during library preparation (Ziegenhain et al., 2017). Though formidable, not unexpectedly these methods have sensitivity limits, with associated transcript absence events (dropouts) that restrict the faithful delineation of cell subtypes and especially overlook low abundant transcripts such as transcription factors, receptors, and signaling molecules that are often pivotal for accurately describing cell processes and fate (Bacher and Kendziorski, 2016; Vallejos et al., 2017). This is a consequence of high abundance transcripts occupying the available NGS read space and is exacerbated by exponential PCR-directed library preparation routines.

Targeted approaches forgo global transcriptome screens, preferring to select transcripts of known utility and are especially favored for mechanistic studies. Diverse targeted strategies have emerged; physical recovery of transcriptome subsets (Riemondy et al., 2019), coupling custom primers to poly (dT) capture beads (DART-seq) (Saikia et al., 2019) and panel selection by PCR as with the Rhapsody workflow (BD) (Salomon et al., 2019). These methods are technically challenging and introduce substantial costs. In order to overcome these limitations, we developed a fast, easy to use, accurate, and highly flexible method for targeted single cell transcriptomics, while imparting extreme sensitivity to overcome data sparsity problems. We call the method Constellation-Seq and demonstrate its power by application to investigations of a specific, rare population of immune cells: dendritic cells (DCs). DCs play a central role in pathogen sensing, phagocytosis, and antigen presentation (Steinman, 2003). Historically DCs have been defined by a combination of morphology, localization, functions, and expression of a restricted set of surface markers (Fromm et al., 2016; Muzaki et al., 2016; Haniffa et al., 2012; Polak et al., 2008). Single cell RNA sequencing technologies have opened the opportunity for in depth investigation and re-defining the classification of these elusive, yet critically important cells. Villani and colleagues redefined the complexity of blood DC populations, describing 6 transcriptomically unique subsets (Villani et al., 2017). However, investigations of their identities and respective roles they play in immune response regulation are limited by their low abundance in tissues and blood. Constellation-Seq enables tracking of the rare DC population without disruptive processing of the PBMCs, expands our knowledge about the prevalence and activation status of sub-populations of blood DCs in health and disease, and presents an attractive diagnostic means linking to future therapeutic strategies.

The limitation of current scRNA-sequencing techniques relates to the difficulty differentiating biologically inactive genes from technical drop-outs, which impact interpretation of the results, can confound normalization, marker selection and more importantly, cell type labeling and the discovery of new cell types. Here, we describe Constellation-Seq, a remarkably simple, inexpensive and scalable (e.g. >200 targets) approach. The method introduces a linear amplification stage in advance of conventional library preparation. Superior performance is demonstrated with two orders of magnitude sensitivity gains for describing system architectures and processes with unprecedented resolution.

**Figure 1:**
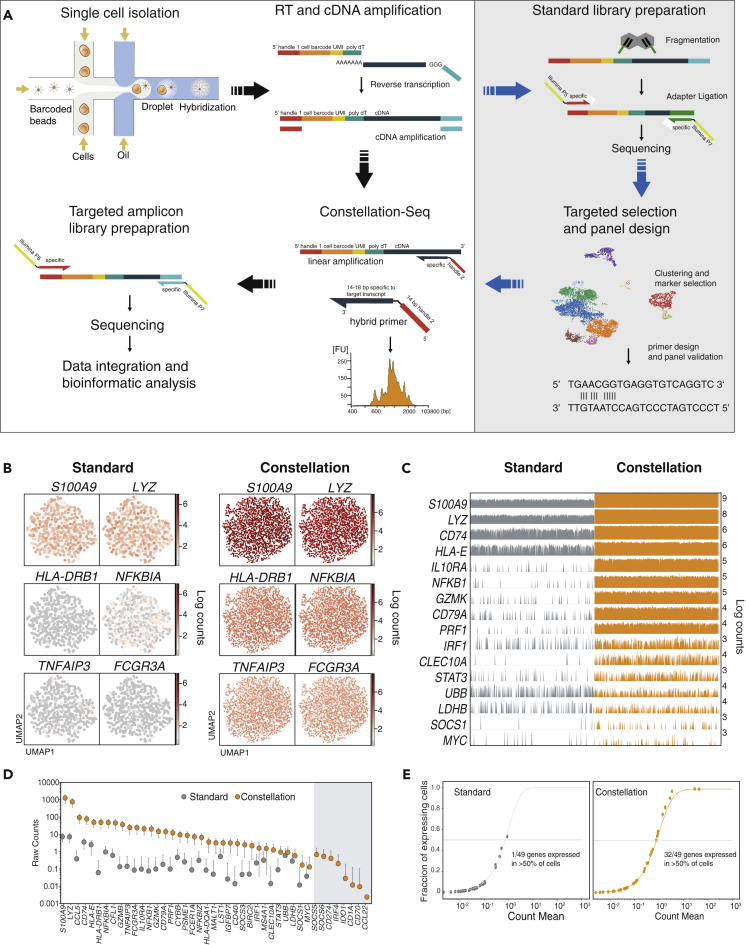
Constellation-Seq methodology and performance (A) Schematic representation of the method: Constellation-Seq can be applied to any Smart-Seq-like library following the standard cDNA synthesis protocol. With a defined primer panel, Constellation-Seq can be applied directly to the cDNA library (black arrows). Otherwise, an aliquot of the cDNA can be used for bulk sequencing and after data analysis the panel of primers can be selected for hypothesis testing or to reduce the technical zeros (Blue arrows). Constellation-Seq includes a hybrid primer (14–18 bp specific sequence, black, adjacent to a common 14 bp handle 2, red) that binds to a specific target sequence in the cDNA library. Linear amplification of 500–1000 bp stretches of target transcripts allows selective enrichment of targets of interest, and the inclusion of the cell barcode and UMI sequences, leads to generation of the Constellation library, ready to use in next-generation sequencing. (B-E) Constellation-Seq was compared against standard sequencing using a panel of 52 targets on control beads. (B) UMAP representation of control beads with standard sequencing compared with Constellation-Seq. (C) A track plot showing the reduction in the data sparsity in a head to head comparison. Each bar represents a gene expression signal from a single cell. A full track plot is included as Figure S4. (D) Individual target raw counts show ~100-fold sensitivity gains for Constellation-Seq, error bars represent SD. (E) Dramatic reduction in technical zeros achieved by Constellation-Seq compared with DropSeq. At 2K UMI counts per bead 32/49 genes were detected in half of the beads (3 negative controls were not detected) using Constellation-Seq, whereas only 1 was detected with the same threshold using DropSeq.

The capture beads each support 10^10^ probes (Saikia et al., 2019) indicating that sensitivity losses arise from the restricted NGS read space (~10^4−6^/cell) and also from exponential PCR amplification during library preparation, where abundant and more efficiently replicated transcripts dominate the available reads. In contrast, linear (single primer) amplification provides an unbiased route to enrichment across transcripts (Hashimshony et al., 2012; Tang et al., 2011). Therefore, in our approach we have used linear amplification following cDNA synthesis for the targeted enrichment of transcripts of interest. The method involves replacing the template switching oligo (TSO) with hybrid primers containing a transcript-specific region adjacent to a universal handle to select and barcode desired transcripts in a single linear amplification. The method is illustrated in Figure 1A and compared with Drop-Seq and other targeted methods in Figure S1). We introduced this linear targeted amplification step to the scRNA-Seq pipeline to provide a direct comparison that is amenable to cost-effective, large-scale cell screening campaigns albeit with recognised sparsity limitations (Ziegenhain et al., 2017; Lähnemann et al., 2020). The panel of primers can be selected based on previous knowledge of the system, from the literature or hypothesis driven. In addition, an aliquot of the cDNA can be used for standard, bulk sequencing from which a group of target genes can be identified and then used for interrogating the same sample at high resolution.

## Results

### Constellation-Seq dramatically reduces the sparsity in scRNA-Seq data

Constellation-Seq was first establish for the DropSeq method and further extended to 10X chromium Single Cell 3′ V3. To exclude biological variation we first used the DropSeq protocol for producing standard beads bearing bulk RNA (Macosko et al., 2015; Svensson, 2019). Using a panel of 20 target genes, sensitivity was compared between single primer linear amplification and dual primer exponential amplification (PCR, requiring an SMART-Seq reverse primer) akin to state of the art methods (*e.g.* Rhapsody, BD) (Salomon et al., 2019). The primer panel contained high, medium and low expression level transcripts specific for peripheral blood mononuclear cells (PBMCs), including markers of newly described blood DC populations (Villani et al., 2017) and activation traits (Table S1). Constellation-Seq is amplification cycle and primer concentration dependent (Figure S2), with straightforward optimisation enabling the selective capture of desired transcripts which produce a characteristically spiny tapestation plot (Figure 1A).

Critically, at 12K reads/bead, linear amplification has a low, 7.6 duplication rate, producing 1,818 UMIs per bead to enable the detection of 17/20 transcripts using a 50% dropout cut-off. In contrast, exponential amplification, at matched depth, has a 33.7 duplication rate, reducing the UMI number to 467 and resulting in only 13/20 transcripts attaining the 50% dropout cut-off. In addition, when the captured UMI were compared, 15/17 genes showed increased sensitivity obtained by linear amplification (Figure S3B).

Next, Constellation-Seq was scaled to 52 targets including 3 negative controls and compared with standard DropSeq (Table S2). Using 15k reads/bead, we demonstrated efficient use of the read space (93.5% reads from target genes) while increasing the average counts/cell 2.7-fold (Figure S3). Constellation-Seq dramatically reduced the degree of sparsity in the data which allows expressed transcripts to be accurately ranked (Figures 1B, 1C, and S4). Individual target transcript counts from Constellation-Seq were on average 83-fold higher. In addition, standard sequencing only detected 41 of the targets, while Constellation-Seq detected all 49 targets and none of the control genes (Figure 1D). The 8 transcripts exclusively detected by Constellation-Seq had average expression levels ranging from 0.03 to 2.60 counts, without length correlation. In practical terms, when using a 50% dropout cut-off, 32/49 are detected by Constellation-Seq and only 1/49 by standard DropSeq at a sequencing depth of 8k reads/bead (Figure 1E). Of merit, the sensitivity of Constellation-Seq cascades directly into significantly lower read requirements; the 32/49 transcripts above 50% cut-off are detected when reducing the depth to 4k reads/bead, with losses (28/49) only evident at 2k (Figure S5). This striking feature of Constellation-Seq presents the option to reduce the sequencing depth and associated experimental cost or increase the scale of the experiment.

### Constellation-Seq reliably measures changes in gene expression

**Figure 2:**
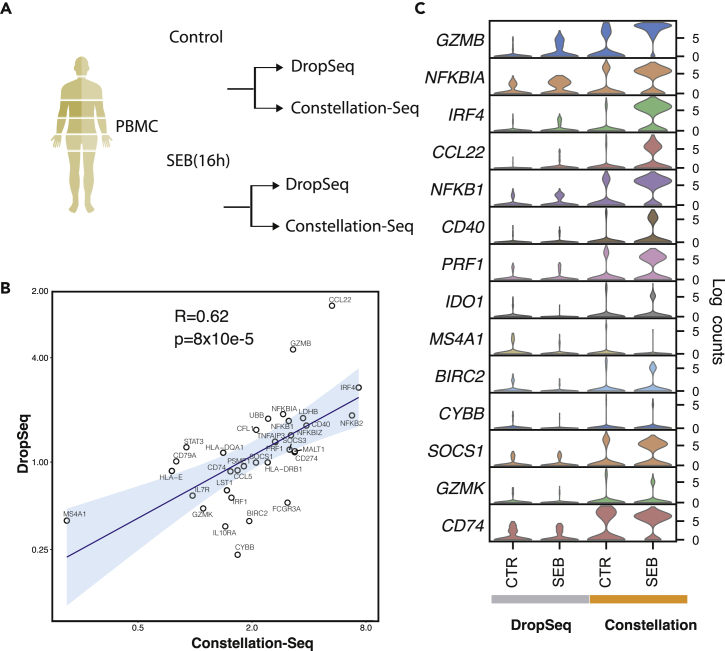
Constellation-Seq reliably measures gene expression changes with higher sensitivity (A) Experimental design; PBMCs from healthy subjects (n = 3) were stimulated with Staphylococcal enterotoxin B (SEB) or media control for 16hr and analyzed using DropSeq and Constellation-Seq. (B) Correlation of normalized gene expression fold changes induced by SEB as detected by DropSeq and Constellation-Seq. Pseudo-bulk counts for each gene used for the comparison. (C) Comparative analysis of selected markers induced by SEB in cultured PBMCs. Violin plots in each row show the distribution and levels of each expressed gene in different culture conditions (CTR – media control, SEB – stimulated cells) and assessed by DropSeq (gray) and Constellation-Seq (orange). y axis represents normalized UMI counts.

To explore the ability of Constellation-Seq to measure gene expression changes in response to perturbation of a cellular system, we challenged human PBMCs with the super antigen Staphylococcal enterotoxin B (SEB, 100 ng/mL, 16 hr). To compare methods 1,000 cells per treatment were sequenced (200K reads/cell for DropSeq and: 20K reads/cell for Constellation-Seq), Figure 2A). In this context, Constellation-Seq consistently detected low copy transcripts such as *GZMB, IRF4* and *SOCS1* with reduced drop-out and increased UMI counts at 10-fold lower sequencing depth. Differential gene expression was compared between control and stimuli for both standard DropSeq and Constellation-Seq. The fold change measurements correlated well between methods (r = 0.62, p value = 8 × 10^−5^, Figures 2B and 2C). Importantly, Constellation-Seq was 1.6 times more sensitive (assessed by the slope of the correlation between Constellation-Seq and DropSeq) to gene expression changes (Figure 2B), improving the resolution of typical activation features such as *NFKB1/NFKBIA* while maintaining comparable expression levels for stable transcripts unperturbed by stimulation (*e.g. CD74*). In summary, the linear amplification step in Constellation-Seq retains the authentic biological response, while measuring responses with greater sensitivity and resolving greater detail in the underlying process.

### Constellation-Seq is compatible with the standard 10x Chromium Single Cell 3′ V3 protocol

**Figure 3:**
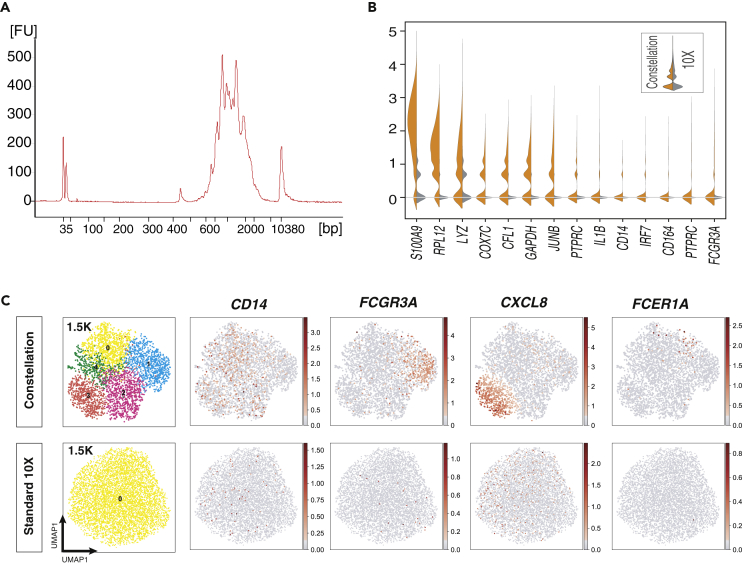
Constellation-Seq is compatible with standard 10-X protocol (A) DropSeq assay of human monocytes (A) The spikes in the plot are characteristic for the Constellation-Seq method due to the selection of targets with distinct molecular weights. (B) Direct comparison of gene expression in monocytes using Constellation-Seq vs 10X. (C) Left: Constellation-Seq (orange) vs 10X (gray), gene expression measured in normalized UMI counts (C). UMAP plot of 6,000 monocyte transcriptomes assessed using 10X and C-10X. At 1.5K UMI counts per cell, C-10X shows more granularity than normal 10X at the same resolution. The enhanced sensitivity of C-10X is demonstrated using monocyte markers.

Next the Constellation approach was reconfigured for use with the popular Chromium 10x Genomics technology using 6,000 CD14 enriched monocytes and amplified cDNA produced using the standard 10X Chromium protocol as the starting material. Following the linear amplification, the library tapestation plot is spiny, typical of targeted transcriptomics (Figure 3A). The targeted library was processed using the Nextera XT protocol. Constellation-Seq greatly improved the detection of transcripts of interest (Figure 3B). Constellation-Seq applied to the 10X library showed 22-fold greater sensitivity allowing reduction of the sequencing depth from 70k to 1.5K reads/cell, while distinguishing 5 clusters, including an activated monocyte sub-population (*CXCL8*). In comparison standard 10X at 1.5K reads/cell failed to resolve these sub-populations and activation states (Figures 3C and S6). Indeed, standard 10X requires 70k reads/cell to obtain the same results, inflating the experimental costs 46-fold (Figure S6) demonstrating both the sensitivity and financial gains achieved using the Constellation-Seq method.

### Constellation- DropSeq can resolve rare DC populations

**Figure 4:**
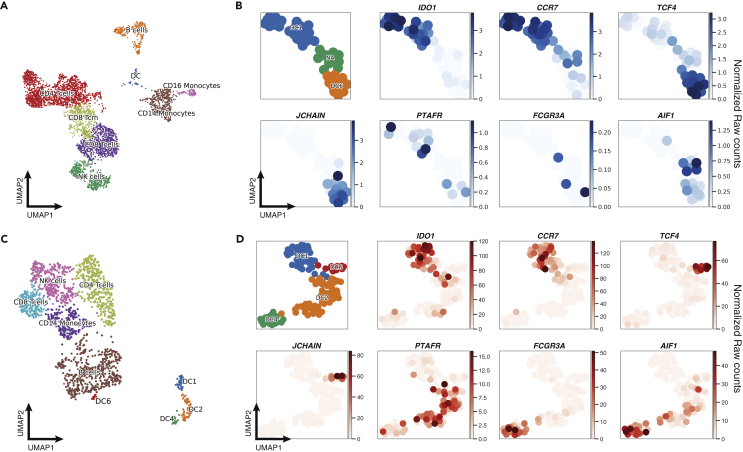
Constellation-Seq can resolve rare cell populations 10X Chromium Single Cell 3′ assay of human PBMC. (A) UMAP projection of 4,182 PBMCs (Leiden r = 0.5, n_pcs = 20, n_neighbours = 20). Cells were grouped into eight clusters. Classification of PBMCs was inferred from the annotation of cluster-specific genes and based on expression of well-known markers of immune cell types using all detected genes. (B) Sub-clustering of dendritic cells (n = 51), from the standard 10X protocol showing marker genes for DC cell populations as in the Villani paper (Villani et al., 2017). (C) Constellation-Seq run of the sample with a panel of 127 genes related to DC biology (n = 1,697 cells). (D) UMAP plot of the dendritic cell subsets (n = 127).

To demonstrate the applicability of Constellation-Seq for the analysis of specific cell subtypes within complex cellular systems, we designed a primer panel targeting 127 transcripts (Table S3) using a recent molecular classification (Villani et al., 2017) for the identification of DC subpopulations and their activation states. 4,000 human PBMCs were processed following the standard 10X Chromium 3′ protocol. While standard sequencing was able to segregate the blood cell types, including DCs and monocytes (Figure 4A), the technique was not sufficiently sensitive to reliably detect all the markers used for identifying DC sub-populations (Figure 4B), limiting the annotations to DC1 and DC6 subtypes. In contrast, the sensitivity of Constellation-Seq allowed the classification of expression markers for four DC subpopulations (DC1: *IDO1*,CCR7, DC2: PTAFR, DC4: *FCGR3A,AIF1,* and DC6: TCF4, JCHAIN (Villani et al., 2017) Figures 4C and 4D). Furthermore, Constellation-Seq provided greater marker detection sensitivity, increasing the average counts by almost 2 orders of magnitude (i.e *IDO1* detection 10X: 0–4 counts per ten thousand (CPTT), C10X: 01–120 CPTTs). The number of DCs detected using Constellation-Seq was substantially higher, 127 vs 51, due to more cells passing the QC filtering and increased clustering achieved by the increase in sequencing depth.

## Discussion

The current sensitivity limits of single cell sequencing methods restrict the scope of biological investigations and impart substantial costs. The simplicity of Constellation-Seq allows inclusion in almost any single cell transcriptome library preparation pipelines involving SMART-Seq primers (DropSeq, Seq-Well, 10X and potentially InDrop). The multiplex scaling capacity is governed by available volume; a 300-plex assay is feasible for a 50 μL reaction volume (without affecting the normal library preparation pipeline; Figure S7). The highly multiplexed selection of transcripts of interest is at the expense of global transcriptome coverage, yet benefits from maximizing the efficient use of the NGS space to enable ultra-sensitive investigations. In this manner, the architecture of cellular systems can be understood with unprecedented resolution and biological processes can be mapped in exquisite detail. Central to Constellation-Seq is prior knowledge of the cellular system, where specific target selection lends strength to mechanistic studies or allows the prioritization of targets for perturbation studies. Additionally, Constellation-Seq can be implemented in drug discovery, delivering preliminary toxicity and efficacy screens for pharmacological compounds of interest. To gain entry to new biological scenarios and to define the targeted primer library for Constellation-Seq, various standard scRNA-seq approaches or bulk transcriptome analyses can first be applied to provide a global screen of the defining molecules and pathways of interest.

Increasing the sequencing depth on informative genes allows specific clusters in the UMAP space to be clearly resolved. In addition, the reduction in technical dropouts allows better cluster labeling, supported on well established surface markers, even at low mRNA expression levels. In addition, it opens the possibility to group the cells based on transcription factors, which may lead to a functional based cell classification. Our experiments exemplify how Constellation-Seq increases the cluster resolution of a population of interest (DCs) in the context of a mixed cell sample (PBMC), where DCs constitute less that 0.5% of the PBMC population (Ueda et al., 2003). We have not only confirmed the expression of markers proposed by Villani et al., falling into the dropout zone in the standard 10X experiment but were able to identify localization of *IDO1* transcript expression specifically to DC1. Importantly, Constellation-Seq resolved the transcriptomic signal of DCs, identifying 127 cells in contrast with 51 detected by standard 10X. This discrimination power is important for validation assays, where the sparsity of marker expression can be misleading for assigning a cell label, and the signal from more abundant cells dominates the population of interest. Application of Constellation-Seq in laboratory settings and in clinical practice will allow tracking of specific cell populations and their activation in health and disease without incurring significant cost.

Constellation-Seq builds on standard scRNA-Seq pipelines, to provide a cost-effective single cell transcriptomics approach for large-scale experiments, while addressing the issues of sensitivity and sparsity. With Constellation-Seq further savings emerge from shrinking the required sequencing depth to allow substantially larger experiments or simply more experiments. Other methods such as Hybridization of Probes to RNA for sequencing (HyPR-seq) and Seq-FISH can be used for targeted RNA detection method. However HyPR-seq requires multiple rounds of washes for probe hybridization and ligation which reduces cell recovery and may affect other genes (Marshall et al., 2020). Seq-FISH, which requires a spatial targeted method, provides an alternative for laboratories with the required infrastructure (Shah et al., 2016). To inform method selection by end users, the experimental economies, including time-finance trade-offs, of Constellation-Seq are compared with standard DropSeq and 10X approaches in Table S4. Beyond this, Constellation-Seq is accessible to resource limited laboratories, overall representing a step toward the democratization of single-cell transcriptomics and the broad-scale expansion of our understanding of biological systems.

### Limitations of the study

A potential limitation of Constellation-Seq is that this approach requires previous knowledge for target gene selection. However, because the method can be used in the same cDNA sample used for standard sequencing, primer selection can be done with the standard pipeline and then Constellation-Seq applied to the same sample. Regarding the multiplex capability, up to now, we have multiplexed primers to detect a total of 127 different genes per single cell. Although this will be adequate for exploring a specific pathway, or cell type, complex samples and hypothesis may require a more extensive gene panels. Based on the concentrations, and current primer design capabilities, the method can be straightforwardly expanded to 300 targets in a single reaction. If more targets are needed, it will be possible to set up more reactions in parallel, with cDNA availability being the limiting factor.

### Resource availability

#### Lead contact

Requests for further information and reagents should be directed to and will be fulfilled by the lead contact Marta E. Polak (m.e.polak@soton.ac.uk).

#### Materials availability

This study did not generate materials than can be shared.

#### Data and code availability

Raw data is available through ENA ENA:PRJEB41830). All notebooks used for bioinformatic analyses are available through GitHub, https://github.com/afvallejo/Constellation-DropSeq.

## Methods

All methods can be found in the accompanying Transparent methods supplemental file.

## Ethics declarations

The PBMCs used in this study were obtained with ethical approval 17/EM/0349.

## Supplemental information

Document S1. Transparent methods and Figures S1–S7

Table S1. Primer panel targeting 20 transcripts, related to figure 1

Table S2. Primer panel targeting 52 transcripts, related to figure 1

Table S3. Primer panel targeting 127 transcripts, related to figure 3

Table S4. Constellation-Seq compared with standard DropSeq and 10-X approaches, related to figure 1
