# Supplementary material for: Resolving cellular systems by ultra-sensitive and economical single-cell transcriptome filtering
Source: iScience. 2021 Feb 5;24(3):102147. doi: 10.1016/j.isci.2021.102147 (PMC7900351; doi:10.1016/j.isci.2021.102147)
Supplement: Document S1. Transparent methods and Figures S1–S7 [file mmc1.pdf]

iScience, Volume *24*

Supplemental Information

Resolving cellular systems

by ultra-sensitive and economical

single-cell transcriptome filtering

Andres F. Vallejo, James Davies, Amit Grover, Ching-Hsuan Tsai, Robert Jepras, Marta E. Polak, and Jonathan West

	DropSeq	Targeted PCR	DART-Seq	Constellation-Seq
mRNA Capture	Poly T	Poly T	Specific Probe	Poly T
Library Construction	TSO dependant	TSO independant	TSO dependant	TSO independant
cDNA amplification	Whole	Targets Only	Targets Only	Targets+ Whole
Type of amplification	PCR	PCR	PCR	Linear

Figure S1. Comparison of Constellation-Seq with DropSeq and other targeted methods, Related to figure 1.
The methods use the same poly T capture probes, with the exception of the DART-Seq method that have probes extended with target-specific capture sequences. For targeted PCR and Constellation-Seq, the library construction will not require the TSO, which can improve the library complexity25. Following mRNA capture DropSeq and DART-Seq methods progress directly to PCR library preparation, whereas targeted PCR and Constellation-Seq methods first involve PCR and linear amplification cycles, respectively.

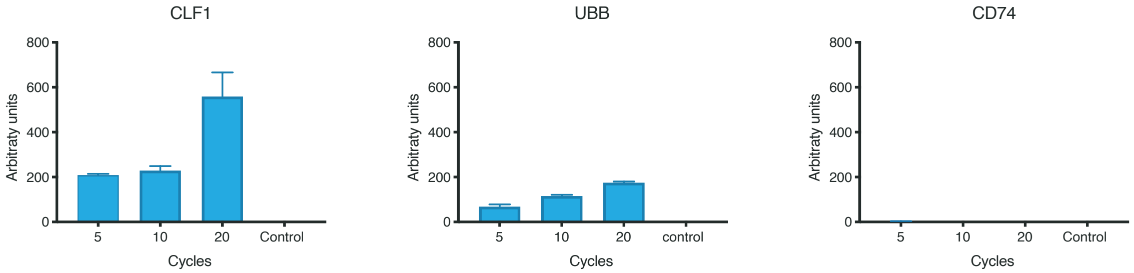

Figure S2 Expression analysis of a Constellation-Seq library containing CLF1 and UBB primers, Related to figure 1.

The library generated from control beads using linear amplification, at a primer concentration of 10 nMol and 65°C annealing temperature was tested with qPCR for expression of CLF1 and UBB as targeted genes and CD74 as a negative control. Data was processed using a semi quantitative approach26. Error bars represent standard deviation (SD).

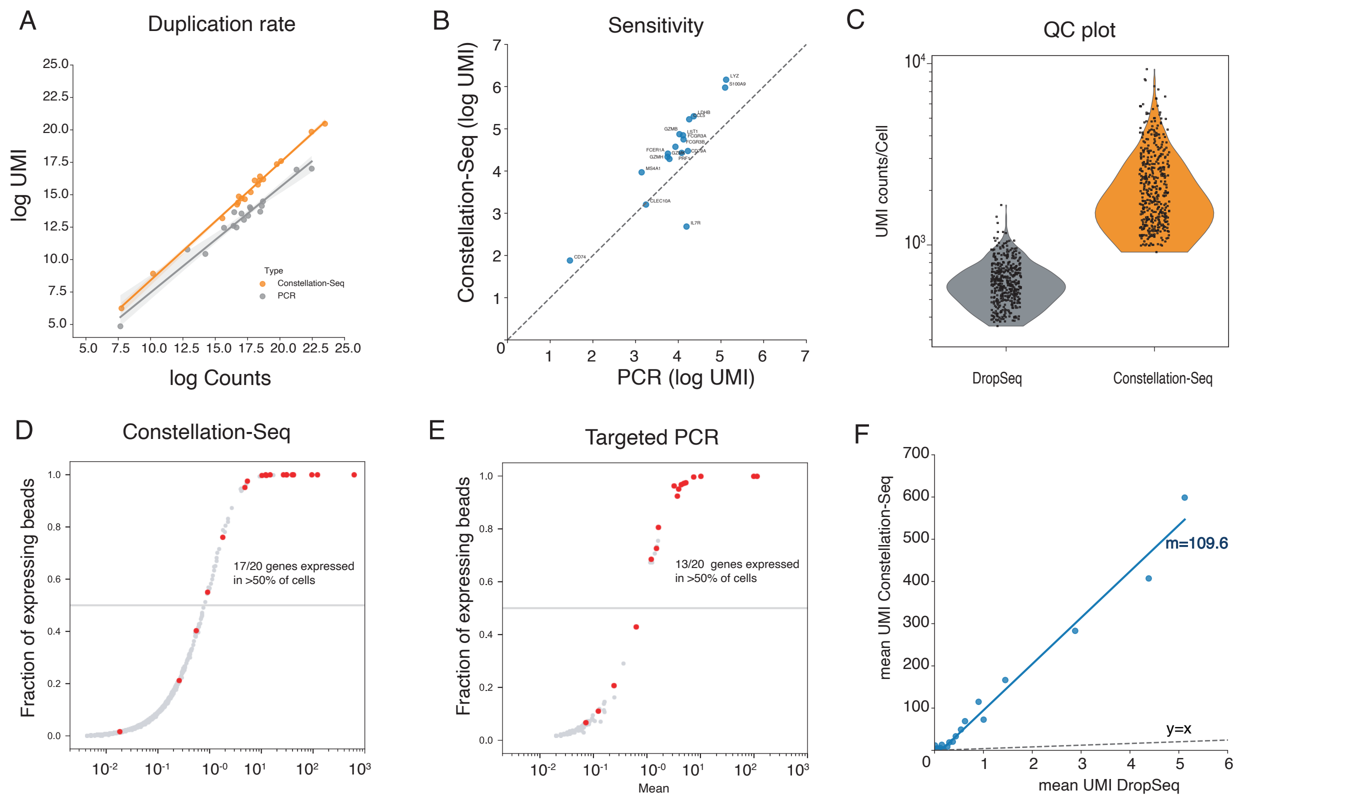

Figure S3 Head to head comparison of detection of 20 targets using linear vs targeted approach, Related to figure 1.

A) Duplication rate analysis at matched sequencing depth. UMI and counts were compared between Constellation-Seq and PCR. The slope was 7.6 and 33.5 for linear amplification and PCR respectively, showing that linear amplification was 4.4 times more sensitive. B) Comparative sensitivity analysis between Constellation-Seq and targeted PCR. Correlation of the UMI captured in both techniques at matched sequencing depth. 15/17 genes above the x=y diagonal demonstrates the increased sensitivity of linear amplification. C) Constellation-Seq counts per bead are 2.7-fold higher than with DropSeq. D-E) Drop-out rate vs mean expression levels in targeted PCR and Constellation-Seq. Red dots represent genes included in the library. F) Comparison of the UMIs captured in DropSeq vs Constellation-Seq.

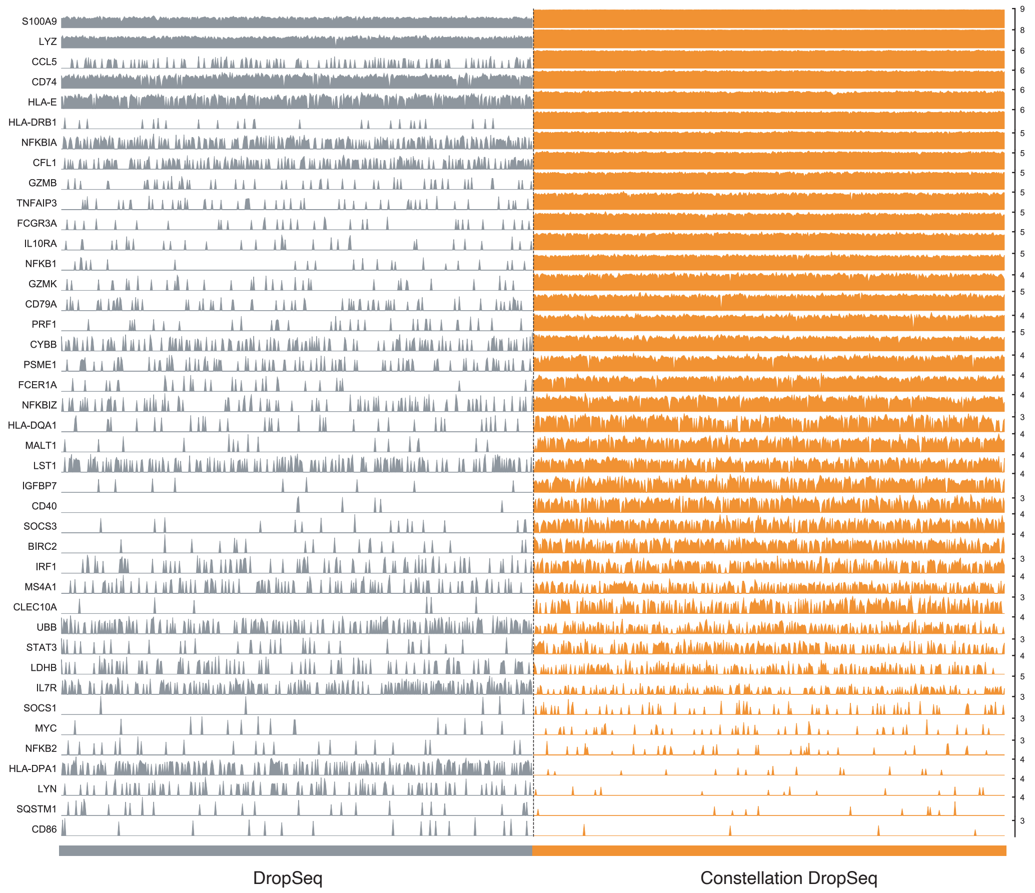

Figure S4. DropSeq and Constellation-Seq comparison for the detection of a panel of 52 genes, Related to figure 1.

A tracksplot of gene expression for high, medium and low expressed genes detected using Drop-Seq (grey) and Constellation-Seq (orange) with control beads. A total of 41/52 genes were detected in both methods. Each bar shows the UMI counts signal from a single cell.

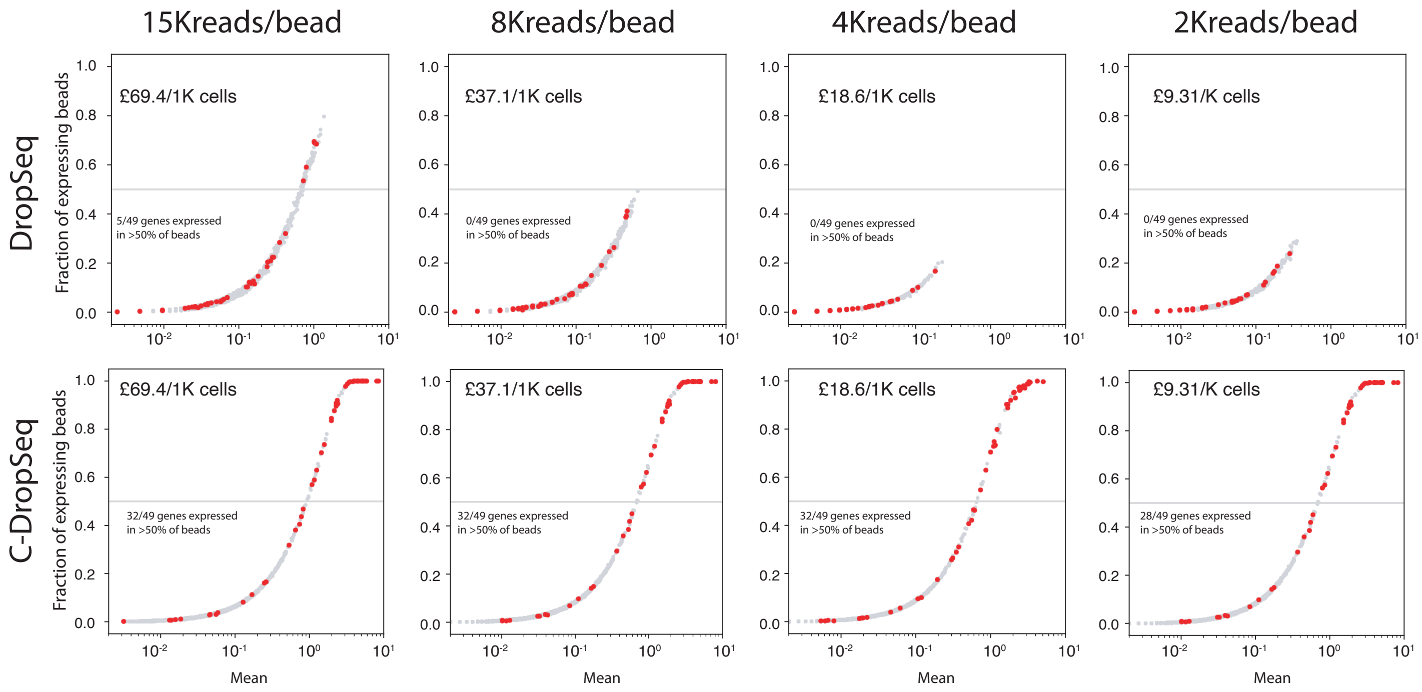

Figure S5 DropSeq and Constellation-Seq sensitivity comparison with varying sequencing depth, Related to figure 1.

The total number of counts for each target was calculated and compared between DropSeq (top) and Constellation-Seq (bottom). The fraction of beads with detected target expression vs mean level of target expression are shown for each gene. The horizontal line indicates the 50% of beads detection threshold. Red: genes from the panel. Grey: genes not included in the panel. Numbers are the predicted effective cost for 1,000 cells.

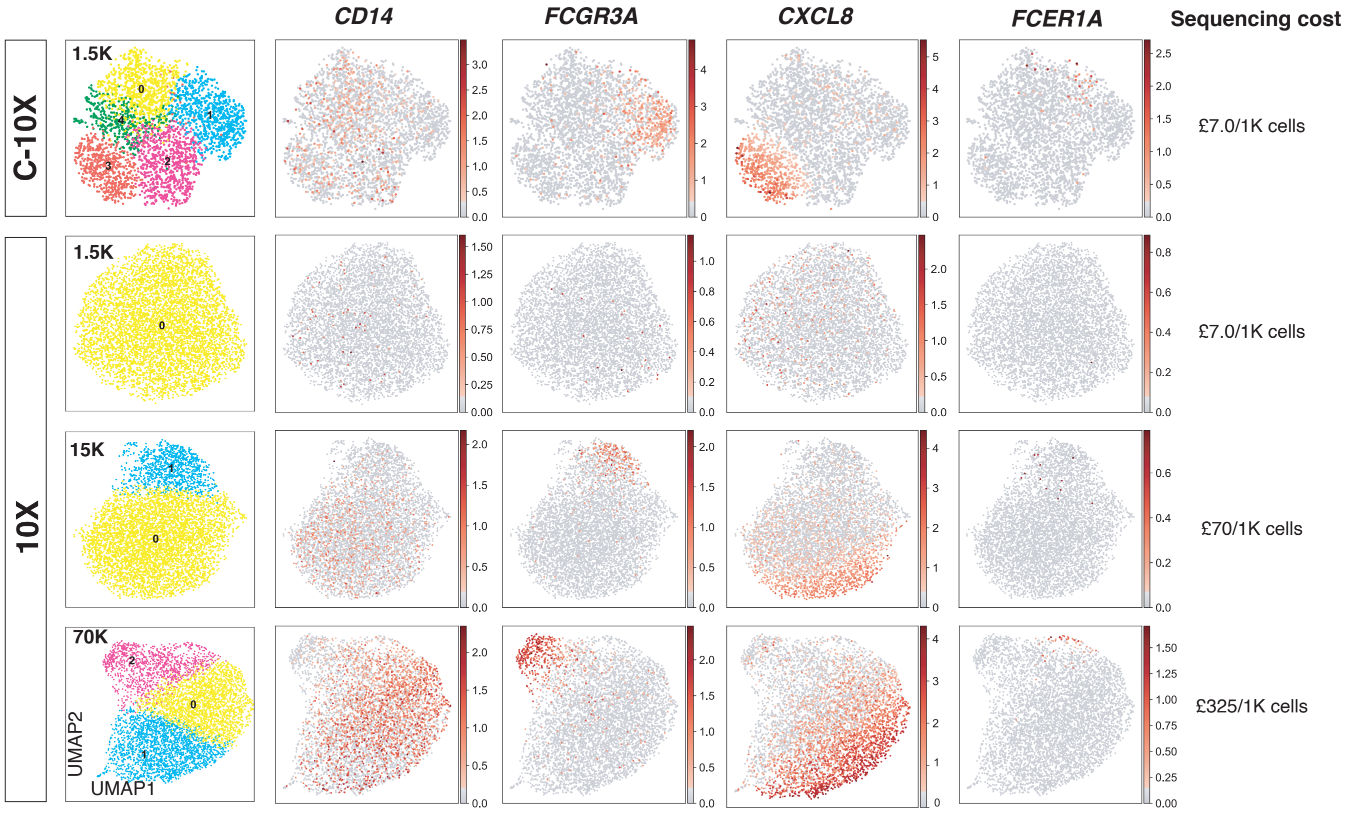

Figure S6 Constellation-Seq can be translated to other single-cell protocols with substantial savings,Related to figure 3.
UMAP plots showing comparison of single cell sequencing of 6,000 monocytes using C-10X at 1,500 reads per cell sequencing depth with standard 10X at varying sequencing depths. Column 1: clustering results, Leiden r=0.5, n_neighbours = 20, columns 2-5: examples of monocyte activation expression markers. Colour denotes gene expression level, as indicated by the legend (normalised UMI counts). Right: the effective cost of sequencing 1,000 cells.

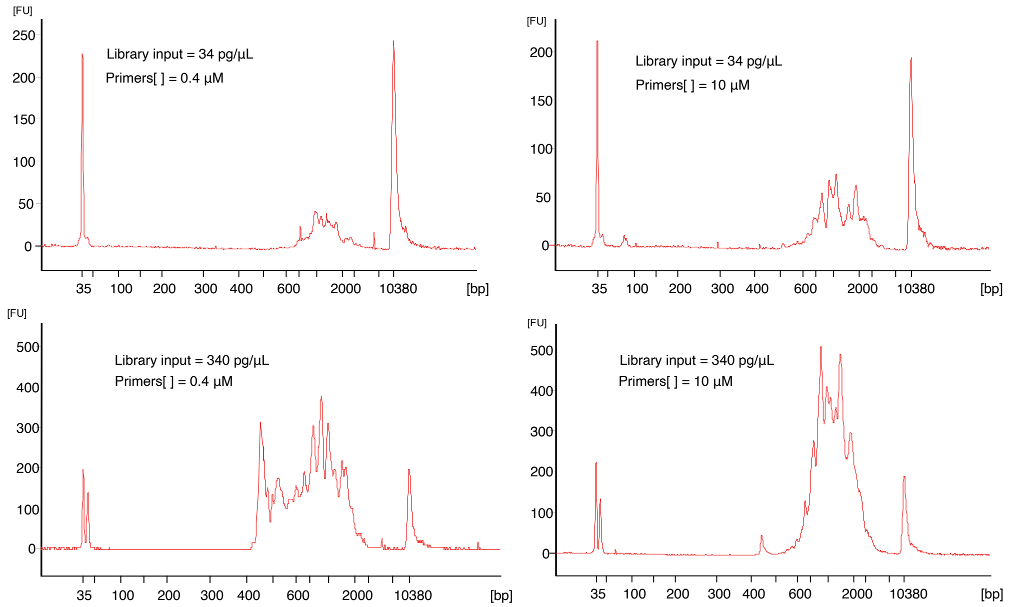

Figure S7 C-10X library optimization,Related to figure 3.
Typical plot from the bioanalyser (Agilent) showing the library input and primer concentration effect on library preparation with C-10X. Top: library input – 34 pg/mL, bottom – library input 340 pg/mL. Left: Primer concentration c= 0.4 µMol, right: Primer concentration c= 10 µMol. Y axis shows fluorescence units (FU) indicating signal intensity and product concentration. The spikes in the plot are characteristic for Constellation-Seq the targeted transcriptomics approach due to the selection of targets with distinct molecular weights.

Transparent Methods

Primer Design

Primers targeting genes of interest were designed using Beacon Designer primer design software (PREMIER Biosoft, California US). The last 14 bases from the SMART primer sequence (TATCAACGCAGAGT) were added to the 5' end of the designed primers. Desired features of primers included: a length between 28-32 base pairs, 40-60% GC content, a primer melting temperature between 52-58°C, and with minimal chance of secondary structures being produced.

Negative control beads

RNA from fresh PBMCs was extracted using RNeasy Plus Mini Kit (Qiagen). Control beads were generated by adding a solution of PBMC RNA at 10 pg/bead, making the RNA content in each droplet equivalent. 200 µL of reverse transcriptase mix (75 µL water, 40 µL Maxima 5x RT buffer, 40 µL 20% Ficoll PM-400, 20 µL 10 mM dNTPs, 5 µL RNase inhibitor and 10 µL Maxima H- RTase) was added to each bead sample. 10 µL of 50 µM TSO was added to the DropSeq controls, whereas for Constellation-Seq no TSO was used. Samples were incubated with rotation at room temperature for 30 minutes followed by 90 minutes at 42°C with continuous rotation. Beads were washed with 1 mL TE-SDS (10 mM Tris, pH 8.0, 1 mM EDTA, 5% SDS) and twice with 1 mL TE-TW (10 mM Tris, pH 8.0, 1 mM EDTA, 0.01% Tween-20). Finally, beads were washed with 1 mL 10 mM Tris pH 8.0, and stored at 4°C.

Cell preparation

Human blood was collected from donors with written consent and ethical approval (study number: 17/EM/0349). PBMCs were extracted immediately using Lymphoprep™ (STEMCELL Technologies) and incubated at 37°C with 5% CO_2. For SEB stimulation experiments cells were cultured in 24 well plates at $2x10^6$ cells/mL for 16h with or without SEB, using a final SEB concentration of 100 ng/mL. For LPS stimulation experiments cells were cultured in 24 well plates at $2x10^6$ cells/mL for 4h with or without

LPS, using a final LPS concentration of 1 μg/mL. Following the incubation period cells were harvested, washed in PBS and counted. 180,000 cells were taken for encapsulation. CD14+ monocytes for the 10X experiment were purchased from Tissue solutions (Glasgow, UK).

DropSeq

DropSeq library preparation and sequencing was performed as described previously(Macosko et al., 2015). Briefly, single cells were co-encapsulated with beads in droplets using the microfluidic design provided by Macosko *et al (Macosko et al., 2015)*. After cell lysis, cDNA synthesis was carried out (Maxima Reverse Transcriptase, Thermo Fisher), followed by PCR (Kapa Hotstart Ready mix, 15 cycles: 4 at 67°C, 11 at 65°C). cDNA libraries were tagmented and PCR-amplified (Nextera tagmentation kit, Illumina). Finally, libraries were pooled and sequenced on an Illumina Nextseq500, (paired end 20x50 bp reads).

Constellation-Seq of DropSeq libraries

For Constellation DropSeq, experiments were processed as normal from encapsulation through to extraction and purification of beads from the droplet emulsion. During reverse transcription however, the template switching oligo (TSO) was absent from the reaction*. This resulted in cDNA fragments without SMART primer binding sites at the 3’ end of the Macosko bead primers. Hybrid primers were pooled at 10 μM. A 50 μL amplification mix was added (25 μL 2X Kapa HiFi Hotstart Readymix, 10 μM primer pool, 24.6 μL water) to aliquots of 2,000 beads (~100 STAMPs). 20 rounds of linear amplification (at 60°C) were first performed before continuing the standard Drop-Seq protocol for library preparation with PCR amplification and tagmentation. cDNA libraries were purified twice using AMPure XP magnetic beads (Beckman Coulter) (1:0.6) and libraries assessed using the Agilent Bioanalyser (KIT) before tagmentation and Next-seq sequencing.

*Standard reagents including the TSO can be used with the caveat of transcript noise generated by the reverse SMART primer.

10x Chromium Single Cell libraries

Single cell libraries were generated using the Chromium Single Cell 3′ library and gel bead kit v3.1 from 10x Genomics. Briefly, 10,000 cells were loaded onto a channel of the 10x chip to produce Gel Bead-in-Emulsions (GEMs). This underwent reverse transcription to barcode RNA before clean-up and cDNA amplification followed by enzymatic fragmentation and 5′ adaptor and sample index attachment using the Nextera XT Library preparation kit (Illumina). Libraries were sequenced on the MiSeq500 (Illumina) with 28x60 bp paired-end sequencing.

Constellation-Seq of 10X Chromium libraries

For Constellation-Seq of 10X libraries, 395 pg of cDNA were used for linear amplification comprising 20 rounds of linear amplification (60°C) using a pool of primers at 40 nM and 0.4 μM of a P5 3'blocked primer. A 40 μL amplification mix was added (20 μL 2X Kapa HiFi Hotstart Readymix, primer pool and P5 blocked primer) to 10 μL of cDNA library. cDNA libraries were purified twice using AMPure XP (Beckman Coulter) magnetic beads (1:0.6) and libraries assessed using a Bioanalyser before tagmentation and Next-seq sequencing on an Illumina Nextseq500, (paired end 28x60 bp reads).

Real Time PCR

Control beads were used to assess the specificity of Constellation-Seq . 400 control beads per well were used as starting material. Constellation-Seq libraries were produced by linear amplification using two control primers (CFL1 and UBB from IDT) for 5, 10 or 20 cycles. Libraries were purified twice using 0.6X AMPure XP magnetic beads (Beckman Coulter) and eluted with 20 μL 1xTE, pH 8.0. Constellation-Seq libraries were tested using specific primers designed within the amplicon region including a negative control, CD74. 2 μL of the Constellation-Seq library was amplified in iTaq™ Universal SYBR (Bio-Rad) containing 200 nM of CFL1, UBB or CD74 primers. Amplification was undertaken in technical triplicates on a HT7900 Fast Real-Time PCR System (Applied Biosystems). Quantification was achieved against a serial dilution calibration curve of the pool of samples in each plate. C_t values were thresholded at 0.1 relative fluorescence units (RFU).

Bioinformatic pipelines

Alignment, read filtering, barcode and UMI counting were performed using kallisto-bustools(Melsted et al., 2019). High quality barcodes were selected based on the overall UMI distribution using emptyDrops(Lun et al., 2019). All further analyses were run using the Python-based Scanpy(Wolf et al., 2018). To remove low quality cells, we filtered cells with a high fraction of counts from mitochondrial genes (20% or more) indicating stressed or dying cells(Macosko et al., 2015). In addition, genes expressed in less than 20 cells were excluded.

Cell by gene count matrices of all samples were concatenated to a single matrix and values log transformed. To account for differences in sequencing depth or cell size UMI counts were normalized using quantile normalization. The top variable genes were selected based on normalized dispersion. This output matrix was input to all further analyses except for differential expression testing where all genes were used.

Visualization and clustering

A single-cell neighbourhood graph was computed on the 50 first principal components that sufficiently explain the variation in the data using 20 nearest neighbours. Uniform Manifold Approximation and Projection (UMAP) was run for visualization. For clustering and cell type identification Leiden-based clustering (Traag et al., 2019) at 0.5 resolution was used. Cell types were annotated based on the expression of known marker genes.

Supplemental References

Lun, A. T. L., Riesenfeld, S., Andrews, T., Dao, T. P., Gomes, T., Participants in the 1st Human Cell Atlas, J. & Marioni, J. C. 2019. EmptyDrops: distinguishing cells from empty droplets in droplet-based single-cell RNA sequencing data. *Genome Biol,* 20**,** 63.

Macosko, E. Z., Basu, A., Satija, R., Nemesh, J., Shekhar, K., Goldman, M., Tirosh, I., Bialas, A. R., Kamitaki, N., Martersteck, E. M., Trombetta, J. J., Weitz, D. A., Sanes, J. R., Shalek, A. K., Regev, A. & Mccarroll, S. A. 2015. Highly Parallel Genome-wide Expression Profiling of Individual Cells Using Nanoliter Droplets. *Cell,* 161**,** 1202-1214.

Melsted, P., Booeshaghi, A. S., Gao, F., Da Veiga Beltrame, E., Lu, L., Hjorleifsson, K. E., Gehring, J. & Pachter, L. 2019. Modular and efficient pre-processing of single-cell RNA-seq. *BioRxiv***,** 673285.

Traag, V. A., Waltman, L. & Van Eck, N. J. 2019. From Louvain to Leiden: guaranteeing well-connected communities. *Sci Rep,* 9**,** 5233.
Wolf, F. A., Angerer, P. & Theis, F. J. 2018. SCANPY: large-scale single-cell gene expression data analysis. *Genome Biol,* 19**,** 15.
